# Circadian Urinary Excretion of Water, and Not Salt, Is Affected by the White Coat Effect

**DOI:** 10.3390/jcm12175725

**Published:** 2023-09-02

**Authors:** Fabio Moretti, Jvan Gianini, Rosaria Del Giorno, Luca Gabutti

**Affiliations:** 1 Faculty of Biomedicine, Università della Svizzera Italiana, 6900 Lugano, Switzerland; 2 Department of Internal Medicine, Clinical Research Unit, Regional Hospital of Bellinzona and Valli, Ente Ospedaliero Cantonale, 6500 Bellinzona, Switzerland; 3 Angiology Service, University Hospital of Lausanne, 1011 Lausanne, Switzerland

**Keywords:** white coat effect, white coat hypertension, hypertension, circadian pattern, circadian rhythm, sodium, sodium excretion, salt, water excretion

## Abstract

Hypertension is an important morbidity factor. The prognostic consequences of the white-coat effect have been studied extensively. The repercussion on the circadian rhythm of urinary water and salt excretion in the same subgroup remain, conversely, among the open topics. Postulating an impaired diurnal sodium and volume excretion we decided to investigate both, in subjects with or without a white-coat effect, in the general population. A sample of 1023 subjects, has been considered. We collected 24-h urine samples, divided in day and night, and we measured the blood pressure with an Ambulatory Blood Pressure Monitoring (ABPM). ABPM values were then compared with physician collected in-office values to assign subjects to the group with or without the white-coat effect. Concerning the circadian pattern of urinary sodium excretion, we found no significant differences between the groups. There was instead in the white-coat effect group a higher night/day ratio of urinary water excretion. The white-coat effect, has been considered a potential hypertension precursor, and its consequent handling could be prospectively relevant in hypertension prevention. The absence of repercussions on the urinary circadian sodium excretion pattern and on the potentially related risk factors in subjects with a white coat effect is reassuring. The clinical significance of the impact on the night/day ratio of water excretion needs to be further investigated.

## 1. Introduction

Hypertension is a common medical condition. The three main components, which boost its prevalence, in addition to the aging of the population, are smoking, the increase in BMI (Body Mass Index), and the change in dietary habits [1,2,3,4].

According to the Swiss health survey on tobacco consumption published in 2017, in Switzerland, 45% of the male population has never smoked, 31% are active smokers and 24% are ex-smokers. In the female population the numbers are a little lower with 23% of active smokers and 19% of ex-smokers [5].

The correlation between BMI and hypertension was already demonstrated by Montani et al. in 2002 [3] and confirmed in further studies [6], with a prevalence of about 20% in subjects with a BMI < 25 kg/m^2^, rising to 50% with a BMI > 30 kg/m^2^ [3].

The main dietary habits that impact hypertension are related to calories and salt intake [1,2,7]. The latter is still underestimated by the population in quantity of consumption, contribution to the development of hypertension and of cardiovascular complications; as was shown by the Swiss survey on salt intake [8]. Even if 81% of the respondents correctly associated increased salt intake with the risk of having high blood pressure (BP), only 41% identified the link with cardiovascular diseases, 21% with myocardial infarction and 21% with stroke. In the same study it was also found that among the 458 participants who reported their salt consumption as being “low” or “very low” only 19.7% (8.3% of men and 28.9% of women) had 24-h urinary salt excretion below the international recommendation of 5 g/24 h [9]. The results confirmed how much unawareness there is about the potential consequences of salt intake in the general population.

In previous studies it has been shown that there is a relationship between circadian rhythms and hypertension, for instance in the review of Bonny and Firsov where they detailed the circadian regulation in the expression of proteins involved in renal sodium homeostasis and blood pressure control [10]. Among the studies cited, Susa et al. demonstrated modifications “in a circadian manner on the posttranslational level”, which was interpreted as a mean to “decrease sodium reabsorption during the active phase and to increase it during the rest phase” [11]. Furthermore, they cited the Doi et al. work demonstrating that the secretion of aldosterone by the adrenal glands is influenced by the circadian clock [12]. The fact that hypertension is strongly related to our circadian rhythm explains also why “accumulating evidence suggests that the antihypertensive medication should be preferentially taken at vesper time” [10]. This suggests that better understanding the blood pressure pattern in its different forms and aspects could help in developing strategies aimed at optimizing blood pressure control and preventing hypertension related damages. 

The consequences of the circadian rhythm of sodium excretion on blood pressure has been recently reviewed by Soliman et al. [13] and investigated, among others [14,15], by our group, demonstrating that, in subjects older than 50 years, impaired daytime sodium excretion capacity is associated with a reduced decrease in nighttime BP, also called non-dipping [16]. We explained the difference, only present in old age, with the related reduction in renal function, the increase in the prevalence of salt-sensitive subjects and the frequency of low-renin essential hypertension. Other studies have also demonstrated that non-dipping patients that have hypertension also show a reduced nocturnal sodium excretion [17,18,19,20,21]. The molecular basis of the transcriptional-translation feedback loop regulating the circadian rhythm of sodium excretion has been summarized in its review by Soliman et al. [13].

In addition, non-dipping nocturnal water excretion has also been associated with the presence of hypertension [22].

Another important determinant of sodium and water renal excretion regulation is the local Sympathetic Nervous System (SNS) activity. It is in fact considered acquired that the Renal Sympathetic Nerve Activity (RSNA) is one of the main contributing factors of pathological increase in renal sodium retention, and that a decrease in urinary sodium excretory ability results in an increase in BP, ending up in activating the pressure-natriuresis mechanism necessary to maintain the sodium balance [22]. The potential consequence of a decrease in renal sodium excretory ability is therefore the development and maintaining of hypertension. This was further confirmed after proving that renal denervation results in preventing the development, mitigating the magnitude, or delaying the onset of hypertension [23].

Di Bona also reminds us that the link between SNS and renal function is not confined to its effect on sodium retention, affecting also Renal Blood Flow (RBF), namely reducing it when activated [22]. The consequences of the activation of the Renal Sympathetic Nerve are however not based on an on/off mechanism, since its different effects are frequency dependent. At lower frequencies of RSNA there is an increase in renin secretion rate, without changes in urinary sodium excretion, RBF, or Glomerular Filtration Rate (GFR). When the frequencies are slightly higher, a urinary sodium excretion decrease accompanies a further increase in renin secretion. At even higher frequencies there is also a reduction in RBF and GFR [22]. 

The correlation between SNS activation and hypertension has been shown both in obese and lean patients, but it is more prevalent in patients having metabolic syndrome and obesity-related hypertension [24]. In the same study, Esler discusses as further proof of the strong association between an increase in RNSA and hypertension the fact that in patients showing a resistant hypertension, ablation of the renal sympathetic nerves lowers BP. Furthermore, during the night, in the sleeping phase, the SNS is less activated, inducing less sodium retention [22].

In 2000, Grassi et al. confirmed the suspected correlation between obesity-related hypertension and sympathetic overactivity [25]. The main mechanism they found responsible is an impairment in the baroreflex-sympathetic modulation, meaning that the increase in BP, measured by baroreceptors, did not translate in the same reflex in obese as it did in lean subjects, resulting in weaker negative feedback. They also found a significant difference in sympatho-adrenergic activity between normotensive and hypertensive obese subjects. A confirmation of this correlation was obtained by showing a decrease in the sympathetic overactivity in severely obese patients after undergoing surgical weight reduction (BMI −9.1 ± 1.4 kg/m^2^) but also with moderate weight loss levels, directly reducing BP [26,27]. 

Lastly, abnormally high cortisol (also known as the “stress-hormone”) levels were also found to be associated with the elevated sympathetic activity in obese patients [28]. These and other correlations, as well as mechanisms, explaining the link between hypertension and obesity were summarized in a 2017 review [26].

The stress related increase in BP, if quantitatively relevant (i.e., in-office Systolic Blood Pressure (SBP) and Diastolic Blood Pressure (DBP) more than 20 and 10 mmHg higher than Home Blood Pressure Measurements HMBP respectively), is called the White Coat Effect/Hypertension [29]. The terms “stress hypertension” are sometimes used as an alternative to white-coat effect because the word “stress” suggests a link to any situation translating in an adrenergic answer. A SNS arousal similar to that induced by the contact with a healthcare professional could in fact derive from many other everyday psychological (relationship conflicts, responsibilities, moving to a new location), physical (sports, hiking, unusual activities) or emotional (grief, fear, pain, etc.) stimuli.

**Figure 1:**
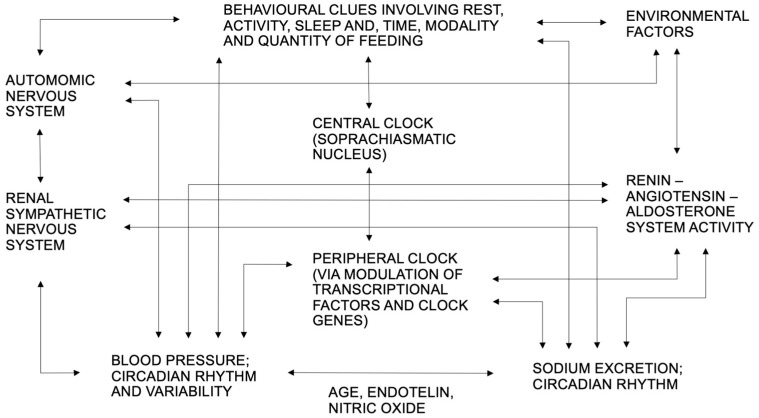
Main actors involved in renal sodium excretion circadian rhythm. Arrows indicate interaction.

Considering the high complexity of the interactions among the actors potentially involved in regulating the circadian rhythm of renal sodium excretion, a non-exhaustive scheme for explanatory purposes is presented in Figure 1.

Whether to treat subjects with a white-coat effect is a matter of discussion and is currently debated. Cuspidi et al. failed to reach a definitive conclusion because there are not enough studies showing the supposed cardiovascular (CV) protective effect of antihypertensive medications in this subgroup of subjects [30].

The risks associated with the white coat effect were however acknowledged in the 2018 ESC/ESH guidelines for the management of arterial hypertension, where it’s suggested to perform, in subjects with the condition, an in-office and out-of-office BP monitoring at least every 2 years [9]. Nevertheless, the treatment that is advised, in patients at low to intermediate CV risk, is mainly directed to lifestyle changes because antihypertensive drugs have shown to be efficient in lowering only office BP without lowering Ambulatory BP (ABP) [31]. Whether the sole reduction of office BP lowers CV risks, as shown in a study where white coat hypertension (WCH) subjects were shown to have the same CV risks as untreated normotensive subjects is still controversial [32]. Mancia et al. suggested that although it has not been demonstrated yet, there are multiple indications that lowering elevated office BP levels, even without altering ABP, has positive effects on long-term CV risk [31]. The main effect obtained with treatment was the reduction of 24-h variability of BP in WCH patients, which has been shown to be an independent predictor of CV morbidity and mortality [32,33,34,35].

In the same guideline, in the table called: “Gaps in the evidence and need for further studies”, further investigating the effects of antihypertensive drugs on white-coat hypertension was considered to be necessary [26].

What we found noteworthy is that, contrary to sustained hypertension where the circadian rhythm of urinary sodium and water excretion has been thoroughly investigated, these determinants of circulating volume regulation in subjects with a white-coat effect are not available.

Given these preambles and the potential repercussions on white coat hypertension management, and postulating an impaired diurnal sodium and volume excretion we are contributing by analyzing the day- and night sodium and water excretion in subjects with and without the condition in the general population.

## 2. Materials and Methods

For the purpose of this study and with the aim of being inclusive, we used the following definitions of White Coat Effect or Stress Hypertension: in-office Systolic Blood Pressure (SBP) more than 15 mmHg higher than Home Blood Pressure Measurement (HBPM) or Diastolic Blood Pressure (DBP) more than 10 mmHg higher than HMBP.

The present study was based on a cross-sectional analysis of a sample of general population residents in southern Switzerland (Canton of Ticino), carried out in 2017 and 2018 called the “Ticino epidemiological stiffness (TEST) study” [16,36,37]. The permanent resident population in Ticino (4.0% of the total Swiss population), consisted of 254,310 Swiss citizens and 97,871 foreigners (accounting for 27.8 percent of the total). Among the latter, the numerically most important nationalities were the Italian (61.9%), Portuguese (7.3%) and German (2.9%). Taking into account the nationalities of the residents and the Swiss cantons of origin (Switzerland is composed of 4 linguistic and cultural regions), the population of Canton Ticino can be considered representative of Central Europe [38]. Recruitment was done by a random sampling from a mailing list that was provided by the Swiss Federal Statistic Department, with inclusion of 1202 respondents (response rate 86%), all Caucasians (0.3% of the total population). It also took into consideration sub-regional differences in habits and lifestyle in the Swiss region under examination and provided a homogenous sample. Informed written consent was obtained and the study was carried out according to the Helsinki Declaration and was approved by the local Swiss ethics committee (CE 3115-2016-01718).

For all subjects studied, a 24-h urine collection, divided in “daytime” and “nighttime”, defined as subject’s self-reported bedtime and wake-up time, was performed, as well as BP measurements by a physician and an Ambulatory BP Monitoring (ABPM), which recorded BP every 30 minutes during daytime and every hour during nighttime. In-office blood pressure was measured using a validated automatic oscillometric device (Dinamap model Pro 100 automated sphygmomanometer, Critikon, Tampa, Florida) and following standardized procedures [39]. The average of the last two of three consecutive BP measurements was considered as the reference value. The ABPM was carried out using a validated, automated oscillometric device (Mobil-O-Graph, I.E.M. GmbH, Stolberg, Germany).

Daytime and nighttime urine collections were analyzed for sodium and creatinine concentrations using a Hitachi equipped with an indirect ion-selective electrode and a Hitachi 717 (both from Roche Diagnostics), respectively.

With the aim of making the measurements comparable and minimizing bias, hourly daytime and nighttime sodium and water excretions were calculated by multiplying the obtained concentration by the volume and dividing the result by the time in hours.

**Figure 2:**
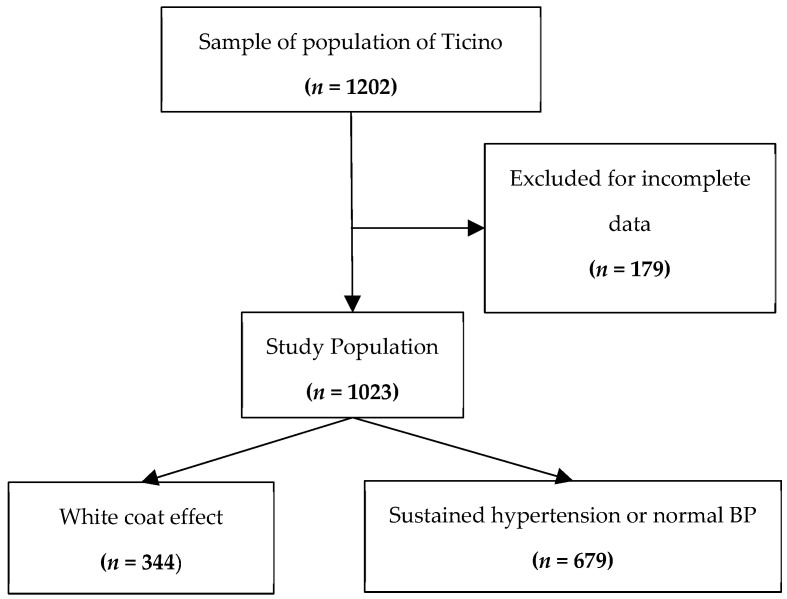
Flowchart showing the participants’ selection procedure.

From the initial population of 1202 subjects, 179 were excluded due to incomplete data. The final number of included subjects was 1023 (Figure 2).

After this first selection we excluded subjects with data extremely out of range (24 h urine volume higher than 5.0 or lower than 0.25 L, 24 h Na excretion higher than 600 or lower than 30 mmol as well as night/day ratio of sodium excretion higher than 10). 

For the statistical analysis we were supported by the CUSSB (Centro Universitario di Statistica per le Scienze Biomediche, Milan, Italy).

Statistical analysis was done based on daytime and nighttime sodium and water urinary excretion per hour according to self-reported bedtime and wake-up time, ABPM results and in-office BP measurements.

The day/night ratio of water and sodium excretion was calculated with the hourly excretion during the night divided by the hourly excretion during the day. The software Statistical Package for Social Science (SPSS) and PHStat add-in, was used for deriving descriptive statistics and for the statistical analysis. The Kolmogorov Smirnov test was used to assess normality. The Mann Whitney non parametric testing procedure was used for comparing the two groups. Logistic multivariate regression was used for assessing the role of covariates. 

## 3. Results

In the population sample (see Table 1 for details), we had 576 (56.3%) females and 447 (43.7%) males. Of these subjects, 559 (54.6%) never smoked, 194 (19%) were active smokers and 270 (26.4%) ex-smokers. Familiarity for arterial hypertension was reported as positive by 465 subjects, whereas 558 denied it. Familiarity for cardiovascular diseases was present in 189 of them, while 834 denied having the risk factor.

To evaluate differences between groups in the distribution of night/day ratio of urinary volume and sodium excretion we used non-parametric tests, since the Kolmogorov Smirnov test showed violation from normality.

**Figure 3:**
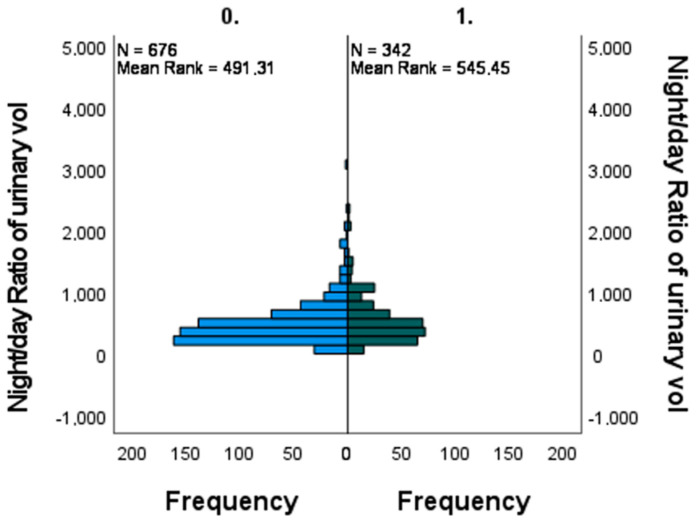
Independent samples Man-Whitney U test showing the distribution of the night/day ratio of water excretion in subjects with (group 1. right) and without (group 0. left) the white coat effect.

**Figure 4:**
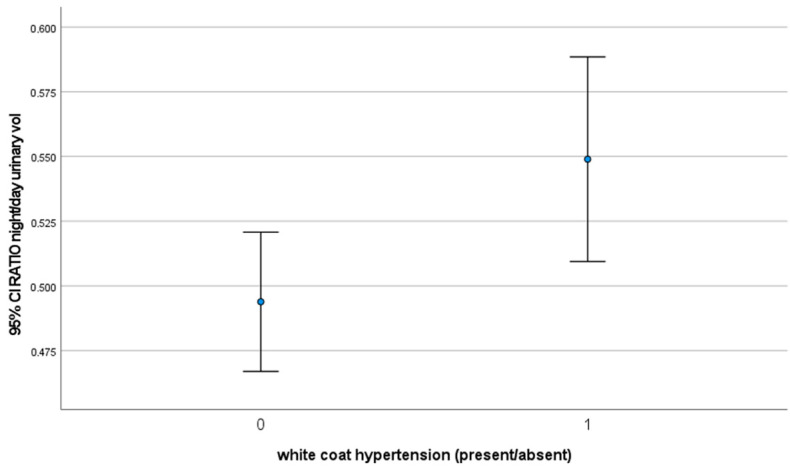
Error bars showing confidence intervals in the night/day urinary volume ratio.

In Table 2, Figure 3 and Figure 4 the results of the Man-Whitney U test are reported for night/day urinary volume. The results suggest that there is a statistically significant difference in the average of the two groups (*p* = 0.006).

The circadian pattern of urinary volume excretion showed a significant difference between subjects with a white coat effect and without; those affected showing a higher night/day urinary water excretion ratio than subjects without the condition.

In Table 3 the results of the Man-Whitney test for night/day sodium excretion are shown. The results suggest that there is no statistically significant difference in the average of the two groups (*p* = 0.106).

**Figure 5:**
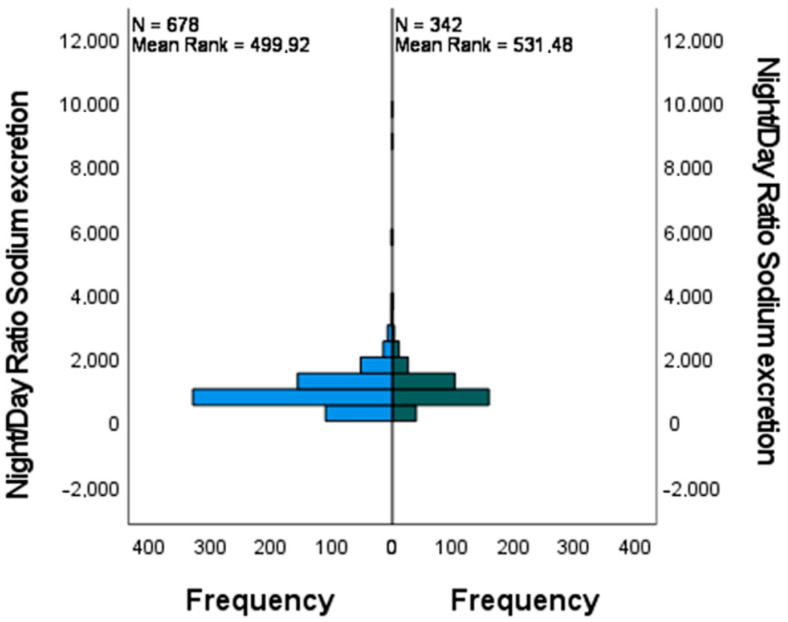
Independent samples Man−Whitney U test showing the distribution of the night/day ratio of sodium excretion in subjects with (group 1. right) and without (group 0. left) the white coat effect.

**Figure 6:**
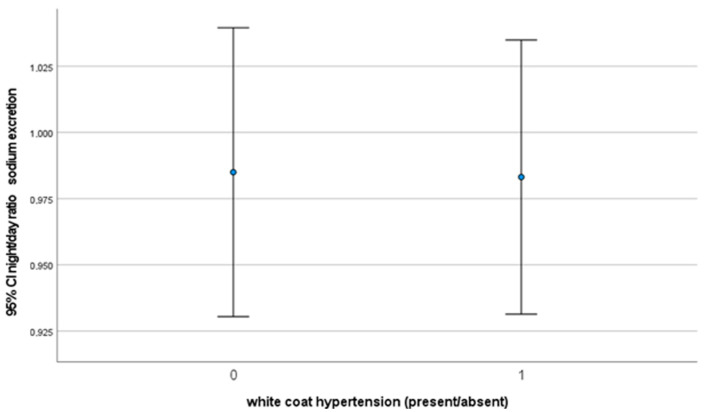
Plot showing confidence intervals in the night/day urinary sodium excretion ratio.

**Figure 7:**
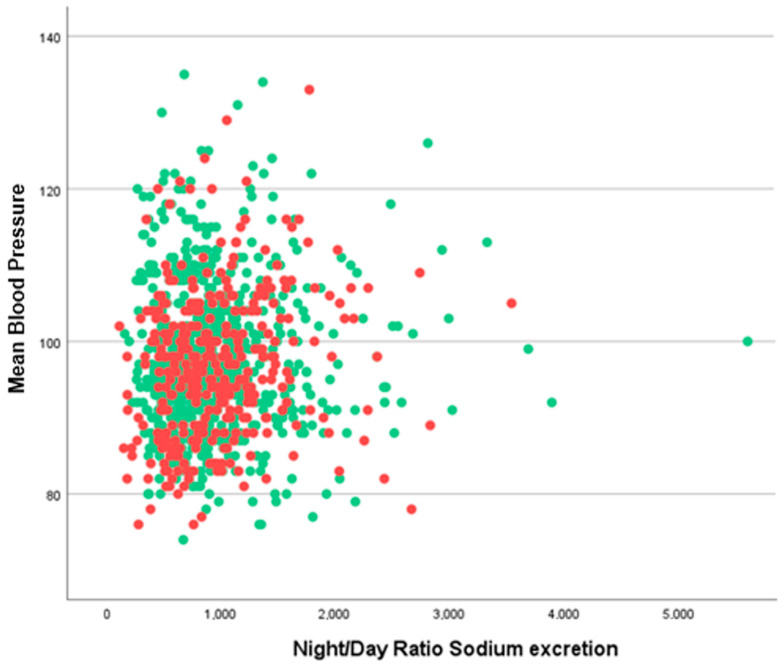
Night/day sodium excretion ratio as a function of mean blood pressure in subjects with white coat hypertension (red dots) and without (green dots).

For the night/day ratio of sodium excretion, we did not find any significant difference, as is shown in Figure 5, Figure 6 and Figure 7 where the confidence intervals at 0.95 level almost overlap.

Last, we investigated, within a multivariate logistic regression, the effect on having a white coat related increase in BP of the most important covariates (age, gender, BP, urine volume and sodium excretion, presence or absence of diabetes and BMI), according to a stepwise variable selection procedure at alpha = 0.05. Age, MAP 24 h and BMI were selected to maximise the likelihood (Table 4).

After removing most of the covariates at a multivariate level (removal prob = 0.05), BMI emerges as a significant risk factor for being in the white coat effect category. 

## 4. Discussion

Previous studies have found a correlation between higher night/day ratio of sodium and potassium urinary excretion and: hypertension (during the night hypertensive subjects excreting more sodium and potassium compared to normotensive) [21,40], higher central BP and Pulse Wave Velocity values [36], and finally a peculiar BP pattern, with higher systolic values during the night and as a consequence a blunt dipping phenomenon [16]. 

A similar analysis in subjects with a white coat effect has not however, to our knowledge, been published. The circadian rhythm of water excretion in hypertensive and white coat effect subjects being in turn not explored in an exhaustive way.

Postulating a stress related impaired diurnal sodium and volume excretion in subjects with the white coat effect we decided to investigate both, in a sample of the southern Switzerland general population.

On the one hand surprisingly, we did not find a mutual influence between the presence of a white coat effect and the night/day ratio of urinary sodium excretion but, on the other we demonstrated a higher nocturnal water excretion in subjects with the condition.

The absence of consequences on the circadian rhythm of urinary sodium excretion is reassuring because it does not add an indirect risk factor for cardiovascular events in subjects with a white coat effect.

As far as water handling, it is difficult to postulate a direct or indirect negative downfall and, with the data we have, it is not possible to distinguish an effect mediated primarily by thirst or by a sympathetic related release of ADH.

Furthermore, considering the observational nature of our cross-sectional study, we cannot draw conclusions about the causality among the observed phenomena. 

Nevertheless, we can hypothesize that both, the absence of consequences on night/day ratio of urinary sodium excretion and the increase in nocturnal water excretion in subjects with the white coat effect could be related to the contextual adrenergic over-activation, which also produces the increase in blood pressure [41]. Postulating that the effect of the sympathetic activation ends up, via a multimodal mechanism, in an ADH related water retention and in a sodium excretion escape while under stress, seems to be the most realistic approach. 

The stress activation of the sympathetic activity and its renal expression involve other pathways among which the Renin-Angiotensin-Aldosterone System (RAAS) and the production of cortisol, which in turn act on the kidney handling of sodium, generating a complex renal tubular answer based on stimuli, counter-regulations and escapes interacting with the circadian rhythm.

The body’s ability to maintain and regain the balance of an electrolyte as critical to homeostasis as sodium, even when put under pressure by stress, is a further confirmation of its proper functioning and the likely absence of major cardiovascular consequences of the white coat effect. 

Concerning the observed relationship between obesity and the white coat effect, in 2009 Ben-Dov et al. found an opposite correlation [42]. An important limiting factor of their study was however the fact that the office BP was measured by a technician, and not by a physician, potentially limiting the white-coat effect detection. They also only included the ABPM values registered during day-time. Their study also cast doubts on the reliability of previous studies such as that of Kotsis et al., where they found a direct correlation between BMI and white coat hypertension assessed by ABPM and non-dipping status [42,43]. An aspect that they took into consideration differently was the definition of “awake” daytime BP, not considering daytime naps and using the 24-h ABPM to define WCH. Independently from the details of data collection and interpretation, previous studies confirmed the correlation between obese patients and WCH that once again was highlighted in our analysis [44,45].

The fact that the prevalence of both, hypertension and the white coat effect is increased in obesity further corroborates the theory that stress related hypertension could be a precursor of sustained hypertension.

Among the limitations of our study, there is a lack of stratification according to the severity of the white coat effect and the presence or absence of a relevant blood pressure variability in the ABPM, due to the narrow sample size. 

Nonetheless, we have been able to analyze at the individual level, using the subject’s self-reported bedtime and wake-up time, the urinary excretion circadian pattern of both sodium and water, and to select subjects with and without white coat effect through a blood pressure assessment done directly by a physician. 

Even if we did not demonstrate consequences on nocturnal natriuresis in the subgroup of subjects with the white coat effect, considering the magnitude of the risk of developing sustained hypertension and related cardiovascular events [46], we suggest to repeat a similar analysis in a group with a higher probability of a sustained sympathetic activation.

## 5. Conclusions

The absence of repercussions on the sodium urinary circadian excretion pattern and on the potentially related risk factors in subjects with a white coat effect is reassuring. The impact on water excretion and its pathophysiological significance needs to be further investigated.

## Figures and Tables

**Table 1:** Participant characteristics and descriptive statistics.

		Age (Years)	BMI (Kg/m^2^)	Systolic BP Office (mmHg)	Systolic ABPM (mmHg)	Diastolic BP Office (mmHg)
N	Valid	1023	1023	1023	1023	1023
	Missing	0	0	0	0	0
Mean		54.17	25.01	130.68	119.09	81.02
Median		54.00	24.40	129.00	117	80.50
Mode ^1^		55	24.50	121.50 ^a^	116	78.50
Std. Deviation		13.61	4.40	17.05	11.84	11.86
Minimum		21	16.30	46.0	90	29.50
Std. Deviation		93	53.20	210.50	179	119.00
Percentiles	25	46.00	21.90	119.50	111	73.50
	50	54.00	24.40	129.00	117	80.50
	75	63.00	27.40	141.00	126	88.00
		**Distolic ABPM (mmHg)**	**Ratio (Night/Day) Hourly Natriuria**	**Ratio (Night/Day) Hourly Urinary Volume**	**Creatinine Excretion Night (mmol)**	**Creatinine Excretion Day (mmol)**
N	Valid	1023	1023	1023	1023	1023
	Missing	0	0	0	0	0
Mean		74.16	1.08	0.64	5.75	8.96
Median		73	0.87	0.43	4.10	7.00
Mode ^1^		72	0.91	0.50	3.15 ^a^	4.60
Std. Deviation		8.77	2.12	2.29	21.82	19.35
Minimum		50	0.12	0.04	0.48	1.20
Std. Deviation		110	58.59	60.35	511.00	603.00
Percentiles	25	68	0.64	0.28	3.15	4.60
	50	73	0.87	0.43	4.10	7.00
	75	80	1.17	0.63	5.46	10.90

^1^ Multiple modes exist. ^a^ The smallest value is shown.

**Table 2:** Results of the Man-Whitney U test for night/day urinary volume ratio between groups.

Parameter	Value
Total N	1018
Mann-Whitney U	127,891.500
Wilcoxon W	186,544.500
Test Statistic	127,891.500
Standard Error	4430.145
Standardized Test Statistic	2.775
Asymptotic Sig. (2-sided test)	0.006

**Table 3:** Results of the Man-Whitney test for night/day urinary sodium excretion ratio between groups.

Parameter	Value
Total N	1020
Mann-Whitney U	123,113.500
Wilcoxon W	181,766.500
Test Statistic	123,113.500
Standard Error	4441.709
Standardized Test Statistic	1.615
Asymptotic Sig. (2-sided test)	0.106

**Table 4:** Multivariate logistic regression. Selected variables.

		Intercept	Standard Error	Wald	Chi-Square	*p*-Value	Odds Ratio
**Step 1 ^a^**	age	0.025	0.005	21.585	1	<0.001	1.026
	MAP 24 h	0.038	0.008	20.826	1	<0.001	0.963
	BMI	0.141	0.018	59.383	1	<0.001	1.151
	Constant	−2.070	0.730	8.041	1	0.005	0.126

^a^ Variable(s) entered on step 1: age, MAP 24 h, BMI.

## Data Availability

Study data are obtainable from the authors upon reasonable request.
